# Calcium homeostasis is required for contact-dependent helical and sinusoidal tip growth in *Candida albicans* hyphae

**DOI:** 10.1111/j.1365-2958.2008.06592.x

**Published:** 2009-01-20

**Authors:** Alexandra Brand, Keunsook Lee, Veronica Veses, Neil A R Gow

**Affiliations:** Aberdeen Fungal Group, School of Medical Sciences, Institute of Medical Sciences, University of AberdeenAberdeen, UK

## Abstract

Hyphae of the dimorphic fungus, *Candida albicans*, exhibit directional tip responses when grown in contact with surfaces. On hard surfaces or in liquid media, the trajectory of hyphal growth is typically linear, with tip re-orientation events limited to encounters with topographical features (thigmotropism). In contrast, when grown on semisolid surfaces, the tips of *C. albicans* hyphae grow in an oscillatory manner to form regular two-dimensional sinusoidal curves and three-dimensional helices. We show that, like thigmotropism, initiation of directional tip oscillation in *C. albicans* hyphae is severely attenuated when Ca^2+^ homeostasis is perturbed. Chelation of extracellular Ca^2+^ or deletion of the Ca^2+^ transporters that modulate cytosolic [Ca^2+^] (Mid1, Cch1 or Pmr1) did not affect hyphal length but curve formation was severely reduced in *mid1*Δ and *cch1*Δ and abolished in *pmr1*Δ. Sinusoidal hypha morphology was altered in the *mid1*Δ, *chs3*Δ and heterozygous *pmr1*Δ/*PMR1* strains. Treatments that affect cell wall integrity, changes in surface mannosylation or the provision of additional carbon sources had significant but less pronounced effects on oscillatory growth. The induction of two- and three-dimensional sinusoidal growth in wild-type *C. albicans* hyphae is therefore the consequence of mechanisms that involve Ca^2+^ influx and signalling rather than gross changes in the cell wall architecture.

## Introduction

The growth direction and behaviour of apically growing cells are determined by interactions that occur between the growing tip and the surrounding environment. While many of the components that establish and maintain cell polarity are highly conserved in apically growing eukaryotic cells, the responses to environmental stimuli are adapted to the life style of the organism, suggesting that sensing and signalling pathways are hard-wired to the intracellular growth machinery in a species-specific manner. An observed feature of anisotropic growth is a tendency to form spiral, sinusoidal or helical curves. Curved growth in tip-growing systems is contact-dependent and observed only during growth on semisolid surfaces. The pronounced clockwise or anticlockwise spiral growth of hyphae emanating from a fungal colony is a two-dimensional growth pattern that has been observed in over 22 species of fungi, including *Aspergillus nidulans* and *Sordaria fimicola* (Madelin *et al*., 1978; Lundy *et al*. 2001) and the oomycete, *Achlya bisexualis* (Trinci *et al*., 1979). The formation of three-dimensional helices by hyphae has been described as right-handed for the aerial hyphae of *Trichophyton* species (Davidson and Gregory, 1937) and the sporangiophores of *Phycomyces blakesleeanus* (Trinci *et al*., 1979). The gravitropic response of primary roots in *Arabidopsis thaliana* can be subverted to form right-handed helices on contact with hard-agar surfaces (reviewed by Migliaccio and Piconese, 2001).

The hyphae of the dimorphic fungal pathogen, *Candida albicans*, produce regular two- and three-dimensional growth forms on semisolid surfaces. Unlike spiral growth around a colony, where the trajectory of growth is permanently offset to one side of the longitudinal growth axis, the trajectory of *C. albicans* hyphal tips oscillates to produce highly regular, two-dimensional sinusoidal waves. *C. albicans* hyphae are also observed to grow as helices on agar and Cellophane, where invariant right-handed helices are formed (Sherwood-Higham *et al*., 1995). Curved growth is thought to involve the displacement of the hyphal apex relative to its axis (Madelin *et al*., 1978; Sherwood-Higham *et al*., 1995) but the molecular mechanisms are poorly understood. Spiral growth cannot be attributed to electrical or gravitational fields (Ritchie, 1960), but the requirement for surface contact-sensing, or thigmotropism, is common to all three growth forms. On hard surfaces such as quartz, *C. albicans* hyphae (grown in 20% v/v serum, 2% w/v glucose) meander slightly but the growing tips are sensitive to obstacles in the substratum. The response to contact with such obstacles is an immediate re-alignment of the hyphal growth axis (Brand *et al*., 2007). This form of thigmotropism is calcium-dependent and is attenuated by blockade of calcium signalling pathways, for example by chelation of extracellular calcium or by deletion of the stretch-activated calcium channel, Mid1. This channel is thought to act as a mechanosensor for external, contact-mediated interactions and may also serve to mark the site of new tip expansion via localized calcium influx. Mid1 is a putative regulator of the voltage-gated calcium channel, Cch1, and together they control calcium influx into the cell (Fischer *et al*., 1997). Calcium ions are an important second messenger in developmental and stress signalling pathways, where a rise in cytosolic calcium activates the calcium-dependent signalling pathway via the phosphatase, calcineurin (Stathopoulos and Cyert, 1997; Onyewu *et al*., 2004) and the calcineurin-dependent transcription factor, Crz1 (Karababa *et al*., 2006). Fungal cells maintain a low cytosolic calcium concentration of ∼100 nM by expelling ions from the cytosol into intracellular organelles, such as the Golgi or the vacuole (reviewed by Cunningham and Fink, 1994) and may potentially also expel calcium ions from the cytosol across the plasma membrane to the exterior. This ensures that relatively low calcium fluxes can result in significant changes in cytoplasmic Ca^2+^ concentration and hence high responsiveness of the signalling pathway. In this study, we asked whether the contact-dependent initiation of sinusoidal growth in *C. albicans* hyphae was dependent on calcium influx and intracellular homeostasis. We find that both the initiation and morphology of sinusoidal curves responded to changes in environmental conditions and we report that, as with other tropic responses of this organism, calcium signalling and homeostasis are required for normal sinusoidal growth.

## Results

### Initiation of sinusoidal growth is dependent on substrate solidity

**Fig. 1:**
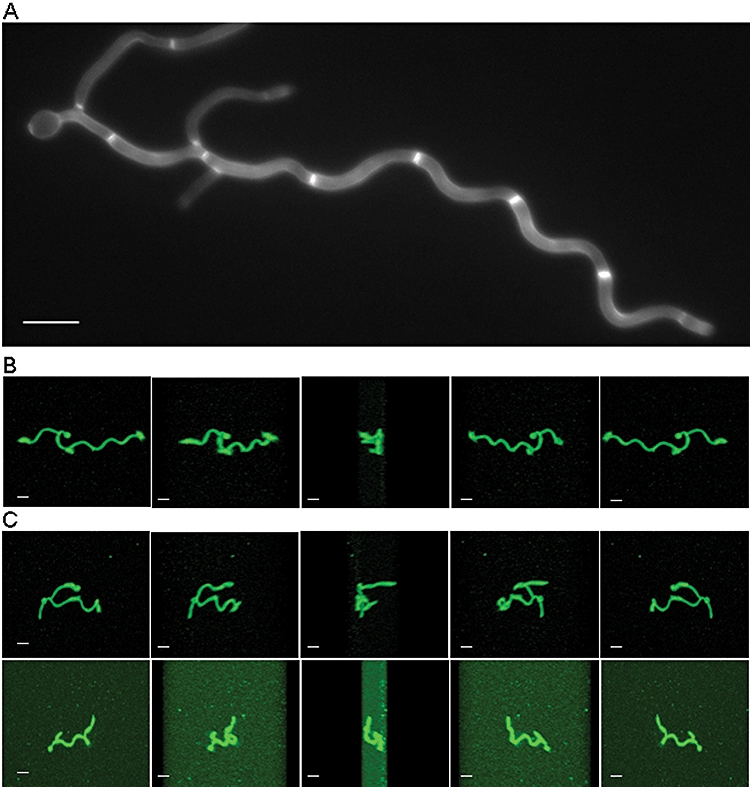
Induction of regular sinusoidal and helical hyphae of *C. albicans* by growth on surfaces in low-nutrient conditions. Sinusoidal curves were induced in hyphae of the control strain by growth on a poly-l-lysine-coated slide in 1% serum. Hyphae were stained with the chitin-specific brightener, Calcofluor White (A). Other hyphae were imaged using scanning confocal microscopy to view hyphal projections that had been stained with the lipophilic dye, FM4-64. Three-dimensional images were constructed and sequential frames are shown for hyphae rotated through the X-plane by 180° (B and C). When grown on 4% (w/v) agar containing 20% (v/v) serum, most hyphae grew as two-dimensional sinusoidal curves that could be shown to be growing in the plane of the substrate when observed as end-on projections (B). However, two examples are shown in C where hyphae formed three-dimensional helical loops that extended above the plane of the substrate. Scale bars = 10 μm.

**Fig. 2:**
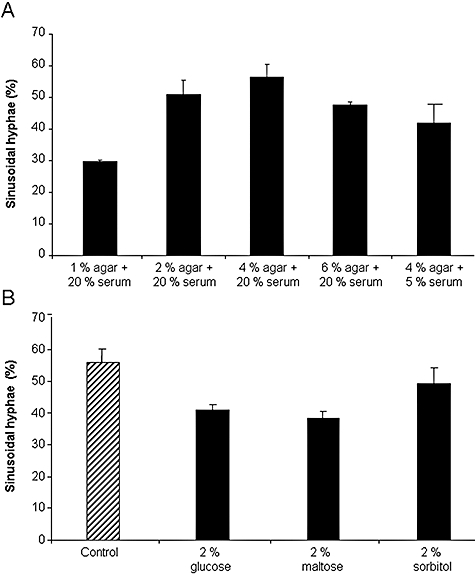
The initiation of curved growth in *C. albicans* hyphae is affected by substrate rigidity (A) or the availability of a carbon source (B). Hyphae were grown on 5% or 20% (v/v) serum and concentrations of agar varied from 1% to 6% (A). Hyphae were grown on 20% (v/v) serum ± 2% (w/v) glucose, maltose or sorbitol at 37°C for 10 h (B). In both experiments, hyphae were viewed by light microscopy and the number of primary hyphae over 80 μm in length that formed two or more consecutive sinusoidal curves was expressed as a percentage of all hyphae formed (error bars are means ± SD, *n* = 100 hyphae from three independent experiments).

*Candida albicans* formed primary hyphae that contained two or more consecutive sinusoidal curves when grown on 20% fetal bovine serum (FBS) solidified with 1%, 2%, 4% or 6% agar (Fig. 1A and B). Helical growth was also observed under these conditions (Fig. 1C). Curve formation on an agar concentration of 1% (w/v) was only half that observed in optimal conditions (*P* ≤ 0.001), demonstrating that hyphae are sensitive to the firmness of the substratum (Fig. 2A). Preliminary experiments demonstrated that the optimal agar concentration for the formation of sinusoidal hyphae was 4% (w/v). On this surface, zones of sinusoidal growth were observed in 57 ± 4.1% (SD; *n* = 3) of wild-type hyphae and subsequently this agar concentration was used in all experiments. On 4% (w/v) agar, lowering the serum concentration from 20% to 5% (v/v) only slightly reduced the number of hyphae producing curves, suggesting that a certain minimal amount of serum is required as an inducer of morphogenesis but serum concentration did not markedly affect sinusoidal growth initiation (Fig. 2A). There were no differences in the propensity to form sinusoidal curves between hyphae of the control strain, CAI4/CIp10, which provides a common genetic background for the deletion mutants used in this study (Brand *et al*., 2004), its wild-type parent, the clinical isolate SC5314 (Fonzi and Irwin, 1993), and a further wild-type strain, 3153 A (Mackenzie and Odds, 1991) (data not shown).

### Carbon source affects sinusoidal growth initiation

It has been observed that sinusoidal growth in hyphae occurs in conditions of limited nutrient availability (Sherwood-Higham *et al*., 1995). To test this further, hyphae were grown in 20% serum supplemented with an additional carbon source in the form of 2% glucose or 2% maltose. To control for the effect of increased osmotic pressure, 2% sorbitol was added as an additional treatment. Sinusoidal initiation decreased significantly in the presence of glucose or maltose (*P* ≤ 0.001) but the presence of sorbitol produced no significant effect. This suggested that the provision of a utilizable carbon source, but not an increase in osmotic pressure, can reduce the onset of sinusoidal growth (Fig. 2B).

### Extracellular Ca^*2+*^ is required for normal levels sinusoidal growth initiation

**Fig. 3:**
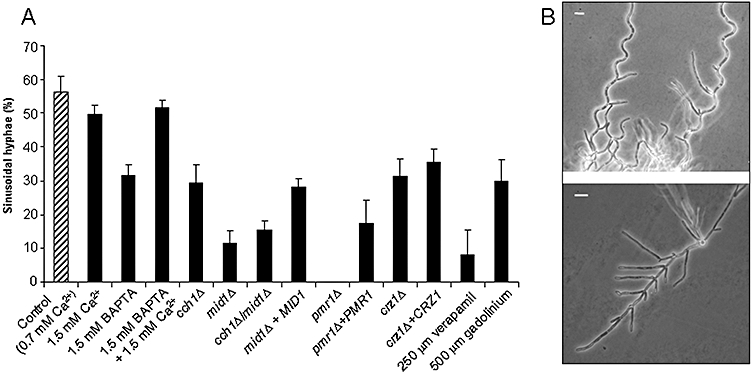
The effect of varying calcium availability and deletion of calcium-related genes on the initiation of sinusoidal growth (A). Hyphal growth was induced on solid agar medium at 37°C and the number of primary hyphae forming two or more consecutive regular sinusoidal waves was determined (error bars are means ± SD, *n* = 100 hyphae from three independent experiments). Light microscopy of hyphae formed by the control strain (B, upper panel) and the *pmr1*Δ mutant (B, lower panel) during growth on 20% (v/v) serum solidified with 4% agar. Bars = 10 µm.

Thigmotropism, or contact-sensing, on hard surfaces in *C. albicans* is attenuated in conditions where extracellular [Ca^2+^] is low (< 5 μM) (Brand *et al*., 2007). To assess whether Ca^2+^ flux is involved in the formation of sinusoidal hyphae, the control strain was grown in conditions of varying Ca^2+^ availability. The [Ca^2+^] in the 20% FBS used in this study was 0.7 mM, consistent with previous findings for Ca^2+^ levels in serum (Blankenship and Heitman, 2005). Sinusoidal growth initiation was not affected by the addition of 1.5 mM Ca^2+^ to serum but was significantly reduced when the Ca^2+^ in serum was chelated using 1.5 mM 1,2-bis(2-aminophenoxy)ethane-N,N,N′,n′-tetraacetic acid (BAPTA) (Fig. 3A). This effect was reversed by supplementation with 1.5 mM Ca^2+^. An accessible supply of exogenous Ca^2+^ is therefore required for maximal initiation of sinusoidal hyphal growth.

### Deletion of channels involved in Ca^*2+*^ homeostasis severely reduces sinusoidal growth formation

Ca^2+^ homeostasis depends on uptake from the environment via plasma-membrane calcium channels, Cch1 and Mid1, and sequestration in the Golgi compartment via the Ca^2+^/Mn^2+^ ATPase, Pmr1 (Antebi and Fink, 1992; Iida *et al*., 1994; Lapinskas *et al*., 1995; Sorin *et al*., 1997; Bates *et al*., 2005; Brand *et al*., 2007). Sinusoidal growth initiation was measured in *cch1*Δ and *mid1*Δ mutants, which have reduced Ca^2+^ uptake, and in the *pmr1*Δ strain, which, in *Saccharomyces cerevisiae*, accumulates abnormally high levels of free cytosolic Ca^2+^ due to constitutive activation of Mid1-Cch1 (Halachmi and Eilam, 1996; Locke *et al*., 2000). Deletion of *CCH1*, which encodes a homologue of the human l-type voltage-gated Ca^2+^ channel, CaV1.2, reduced sinusoidal growth formation by 50%, a similar level as that observed for Ca^2+^ chelation (46%) (Fig. 3A). Cch1 is thought to be regulated by the putative stretch-activated Ca^2+^ channel, Mid1, which acts as a key mechanosensor of topographical changes during the thigmotropic response (Brand *et al*., 2007). Deletion of *MID1* resulted in a more severe phenotype than deletion of *CCH1* because sinusoidal growth initiation in *mid1*Δ and in the *mid1*Δ/*cch1*Δ double mutant was reduced by approximately 80% compared with the control strain (*P* ≤ 0.001) (Fig. 3A). In addition, the morphology of the sinusoidal curves formed by *mid1*Δ mutants differed to that of control cells (see below). Sinusoidal growth initiation was also severely reduced in the presence of verapamil, a blocker of voltage-gated calcium channels (Hockerman *et al*., 1997), and, to a lesser degree, the presence of gadolinium, a blocker of stretch-activated calcium channels in mammalian cells (Caldwell *et al*., 1998). No sinusoidal growth was observed in the *pmr1*Δ strain, where the primary hypha and the branches grew straight, in a rod-like fashion (Fig. 3B). Re-integration of one copy of *PMR1* partially rescued this phenotype, but sinusoidal growth formation was nevertheless 65% lower than the control strain (*P* ≤ 0.001) (Fig. 3A).

The *pmr1*Δ-like, high cytosolic [Ca^2+^] phenotype was ameliorated in *Ustilago maydis* and *S. cerevisiae* by decreasing or increasing the availability Ca^2+^ respectively (Halachmi and Eilam, 1996; Adamiková*et al*., 2004). When hyphae of the *C. albicans pmr1*Δ mutant were grown in the presence of 250 μM verapamil together with 500 μM gadolinium in order to reduce Ca^2+^ entry into the cell, the hypha elongation rate of *pmr1*Δ was reduced but the hyphae retained their markedly straight morphology (data not shown). In *S. cerevisiae*, supplementation of the medium with calcium (10 mM) or manganese (250–420 μM) ameliorated *mid1*Δ or *pmr1*Δ mutant phenotypes (Antebi and Fink, 1992; Iida *et al*., 1994; Dürr *et al*., 1998). Similarly, supplementation with 10 mM CaCl_2_ or MnCl_2_ rescued loss of yeast viability at stationary phase in the *C. albicans pmr1*Δ mutant (Bates *et al*., 2005). Formation of sinusoidal hyphae of the control strain, *mid1*Δ/*cch1*Δ and *pmr1*Δ, was tested in the presence of 10 mM Ca^2+^ or Mn^2+^. Morphogenesis was repressed in all three strains grown in 10 mM Mn^2+^, while in 10 mM Ca^2+^ morphogenesis was observed but hypha elongation was inhibited. When [Mn^2+^] and [Ca^2+^] were reduced to 100 μM and 3.5 mM respectively, hypha elongation was partially restored in the mutants. In the control strain, hyphal length was fully restored but hyphae meandered (data not shown). As sinusoidal growth in the control strain was not affected by the addition of 1.5 mM Ca^2+^ (Fig. 3A), the limiting extracellular [Ca^2+^] for helix formation must lie between 1.5 and 3.5 mM. In summary, the reduction of Ca^2+^ uptake using channel blockers or supplementation of the growth medium with Ca^2+^ or Mn^2+^ did not rescue the mutant phenotype of *pmr1*Δ but did perturb sinusoidal hypha formation in the control strain. The phenotype of the *mid1*Δ/*cch1*Δ mutant was similarly unaffected by Ca^2+^ or Mn^2+^ supplementation.

### Sinusoidal growth and the calcium signal transduction pathway

In fungi, increased levels of cytosolic calcium activate the calcium signalling pathway via the phosphatase, calcineurin, and its transcription factor, Crz1 (Stathopoulos and Cyert, 1997; Onyewu *et al*., 2004; Karababa *et al*., 2006). Deletion of either calcineurin subunit, the catalytic Cmp1 or the regulatory Cnb1, in *C. albicans* results in hypersensitivity to calcium in serum (Blankenship and Heitman, 2005). In accord with these findings, neither mutant formed hyphae on 20% serum in this study. Deletion of *CRZ1*, which encodes the calcium-dependent transcription factor that is activated by calcineurin, reduced sinusoidal hypha formation by 46%, to a similar level as that seen for *cch1*Δ (50%) (*P* ≤ 0.001) (Fig. 3A). The transcription of genes that are controlled by changes in cytosolic calcium levels via the calcineurin–Crz1 signalling pathway may therefore contribute to surface sensing or sinusoidal hypha formation.

### Cell wall composition and sinusoidal hypha initiation

**Fig. 4:**
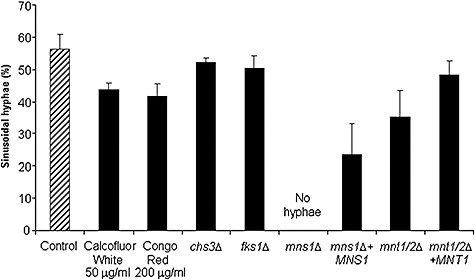
The effect on curved hypha formation of cell wall-perturbing reagents or deletion of genes involved in cell wall biosynthesis or protein mannosylation. Hyphal growth was induced on solid agar medium at 37°C and the number of primary hyphae forming two or more consecutive sinusoidal waves was determined (error bars are means ± SD, *n* = 100 hyphae from three independent experiments).

In addition to high intracellular calcium levels reported in *S. cerevisiae* (Halachmi and Eilam, 1996), the phenotype of the *pmr1*Δ mutant in *C. albicans* also results in changes in the composition of the cell wall due to altered surface mannosylation. The outer wall glycoproteins of *pmr1*Δ have severely truncated *N-* and *O*-linked mannans, accompanied by a compensatory increase in the percentage of the cell wall that is comprised of glucan (Bates *et al*., 2005). The *pmr1*Δ mutant is also hypersensitive to various cell wall perturbing agents, despite the constitutive activation of the cell wall integrity pathway in this strain. To determine whether sinusoidal growth was dependent on normal cell wall structure, initiation was measured in mutants with altered levels of chitin, β-glucan or mannosylation. In addition, control cells were treated with reagents that weaken cross-linking within structural wall polymers, resulting in altered wall integrity. Both the *chs3*Δ mutant, which has an 85% reduction in lateral wall chitin (Bulawa *et al*., 1995), and *fks1*Δ, a glucan synthase mutant which has a 50% reduction in cell wall glucan levels and a threefold increase in chitin content (Mio *et al*., 1997; Walker *et al*., 2008) (Fig. 4), initiated normal sinusoidal hyphal curves. Wild-type cells were grown in the presence of 200 mg ml^−1^ Congo Red or 50 mg ml^−1^ Calcofluor White, which disrupt hydrogen bonding between polymeric chains of β-glucan and chitin, respectively. This disruption reduced sinusoidal hypha formation by 30% (*P* = 0.001) and 25% (*P* = 0.009), respectively (Fig. 4).

Because both *N*- and *O*-mannosylation were affected in the *pmr1*Δ mutant, we attempted to distinguish whether truncation of one or other glycan correlated with the loss of helix formation. To examine the effect of *O*-mannan truncation, we tested the *mnt1*Δ*/mnt2*Δ double mutant, where *O*-linked mannans are truncated at Man_2_ (Munro *et al*., 2005). To test *N*-mannan truncation, we used the *mns1*Δ strain, where reduced Golgi α-mannosidase activity results in the truncation of outer-chain *N*-glycans and loss of phosphomannan (Mora-Montes *et al*., 2007). Helicity was reduced by 34% when *O*-mannan was truncated (*P* ≤ 0.001) (Fig. 4) and this phenotype was rescued by the re-integration of *MNT1*. Although the *mns1*Δ mutant was reported to form normal hyphae in liquid 20% serum, the mutant did not form hyphae on solid 20% serum. In the *MNS1* re-integrant strain, which has a single copy of *MNS1*, morphogenesis was normal and helicity was partially restored (Fig. 4). Similar results were observed for the *mns1*Δ/*MNS1* heterozygous strain (data not shown). Taken together, these results suggest that altered mannosylation status, particularly the lack of outer-chain elaboration of *N*-linked structures, affected helix formation to a greater extent than the disruption of chitin and β-glucan.

### Regulation of helix morphology

**Fig. 5:**
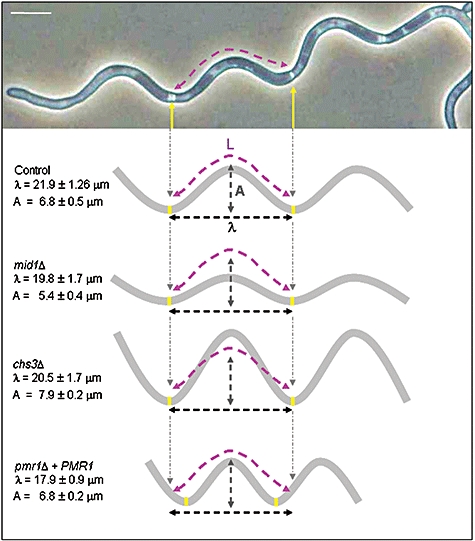
Variation in the morphology of sinusoidal curves in gene deletion mutants compared with the control strain. Hyphal growth was induced on 4% solid agar medium at 37°C and the wavelength (λ) and amplitude (A) of sinusoidal waves were determined using Openlab software (means ± SD, *n* = 100 helices in three independent experiments). Bar = 10 μm.

Each sinusoidal hypha generally consisted of one cell compartment (Fig. 5). The morphology of hyphal helices was characterized by measuring the amplitude (A) and wavelength (λ) of > 100 sinusoidal compartments per strain for hyphae that were longer than 80 μm. For the control strain, the mean values for wavelength and amplitude were 21.9 ± 1.26 μm and 6.8 ± 0.5 μm respectively (mean ± SD, *n* = 100). A variety of environmental conditions, such as agar concentration, and isogenic mutations affected the propensity to form sinusoidal hyphae, but did not affect the morphology of the curves that were formed. The exceptions were mutants with deletion of *MID1* (*mid1*Δ, *mid1*Δ*/cch1*Δ), *CHS3* or strains that were heterozygous for *PMR1* (*pmr1*Δ/*PMR1*, *pmr1*Δ+*PMR1*). In the *mid1*Δ and *chs3*Δ mutants, wavelength (λ) was unaltered compared to control hyphae but the amplitude (A) was lower in *mid1*Δ strains (*P* = 0.008), and higher in the *chs3*Δ mutant (*P* = 0.017). In contrast, the amplitude of the *pmr1*Δ+*PMR1* re-integrant strain was the same as that of control strain, but the sinusoidal compartments were of shorter wavelength (*P* = 0.02). To compare the straight *pmr1*Δ hyphae further with the control strain, the mean cell length (distance between septa) and hypha length were determined after 8 h growth. In the control strain, there was no difference between sinusoidal and straight hyphae in cell compartment length or total hyphal length after 8 h growth [28.4 ± 0.76 μm and 136 ± 24.2 μm respectively (SD; *n* = 100)]. In the *pmr1*Δ mutant, cell length and hypha length were significantly shorter at 14.8 ± 0.89 μm (*P* = 0.03) and 78 ± 0.11.5 μm (*P* = 0.33) respectively.

## Discussion

Growing *C. albicans* hyphae respond to external stimuli such as electric fields (galvanotropism) and changes in substrate topography (thigmotropism) by re-orienting their growth axis in a calcium-dependent manner (McGillivray and Gow, 1986; Crombie *et al*., 1990; Lever *et al*., 1994; Watts *et al*., 1998; Brand *et al*., 2007; 2008). Thigmotropism is thought to rely on the presence of the putative stretch-activated plasma-membrane calcium channel, Mid1, which may act as a mechanosensor of external obstacles and changes in surface topography. We have proposed that activation of Mid1 allows localized calcium influx to determine a new site for hyphal extension by causing an asymmetry in the tip-high calcium gradient observed in many apically extending polarized eukaryotic cells (Brand *et al*., 2007). Chelation of extracellular calcium or deletion of *MID1* severely curtailed the ability of hyphal tips to respond to contact-dependent stimuli. The formation of regular sinusoidal curves and helices is a further example of a contact-dependent directional response observed in hyphal tips, because it is induced by growth on semisolid surfaces. In this study, we show that the factors that are important for thigmotropism are similarly involved in helix formation. However, unlike galvanotropism, which was heightened by the elevation of extracellular Ca^2+^ concentration, thigmotropism and sinusoidal hypha formation were not enhanced by this treatment. In contrast, chelation of extracellular calcium reduced the response of hyphae to all three tropic stimuli. Calcium chelation, deletion of *CCH1* or the calcium-dependent transcription factor *CRZ1*, or the addition of gadolinium, a blocker of stretch-activated calcium channels, reduced the formation of helices to a similar extent. The reduction in helicity was more severe in the *mid1*Δ mutant than the *cch1*Δ mutant. This is consistent with the more pronounced attenuation of thigmotropism of this mutant relative to that in *cch1*Δ (Brand *et al*., 2007) and suggests a key role for this protein in transducing surface-dependent environmental signals.

Mutations and treatments that affected the skeletal components of the cell wall had a relatively small effect on the induction of sinusoidal growth. In contrast, this was completely abolished by deletion of *PMR1*, which encodes a Golgi-located Ca^2+^/Mn^2+^ ATPase that supplies cofactors for glycosidases involved in glycan maturation (Bates *et al*., 2005). The *PMR1* deletion resulted in alterations in intracellular cytoplasmic Ca^2+^ distribution (Halachmi and Eilam, 1996), and the truncation of *N*- and *O*-linked mannans, which in turn can result in misfolding or mislocalization of cell surface proteins (Gibson *et al*., 1981; Machamer and Rose, 1988; Bates *et al*., 2005). We attempted to distinguish whether the dramatic loss of sinusoidal growth in *pmr1*Δ was due to perturbation of calcium ion homeostasis or alterations to glycosylation by testing mutants with specific *N*- or *O*-mannosylation defects. Disruption of *N*-linked mannan had a pronounced effect on the propensity to form sinusoidal hyphae. The localization of Mid1 at the plasma membrane is dependent on *N*-mannosylation in *Saccharomyces cerevisiae* (Ozeki-Miyawaki *et al*., 2005) and the *mid1*Δ mutant was markedly compromised in its ability to initiate sinusoidal growth. Therefore, the straight hyphae observed in the *pmr1*Δ mutant may be a pleiotropic phenotype due to alterations in both the cell surface of the hyphae and the calcium signalling required for contact-sensing. In *S. cerevisiae*, some *pmr1*Δ and *mid1*Δ phenotypes can be rescued by supplementation with Mn^2+^ or Ca^2+^ (Antebi and Fink, 1992; Loukin and Kung, 1995; Dürr *et al*., 1998). In *C. albicans*, 10 mM Mn^2+^ or Ca^2+^ rescued stationary phase viability in *pmr1*Δ (Bates *et al*., 2005). However, we were unable to restore sinusoidal growth in *pmr1*Δ by supplementation with various concentrations of these cations. Taken together with our observation that perturbation of the cell wall had a less significant effect on the initiation of curved growth, it is likely that addition of extracellular ions further perturbed the intracellular calcium gradient required for normal polarized growth. Similarly, supplementation did not restore the *mid1*Δ/*cch1*Δ phenotype, which is consistent with previous findings (Brand *et al*., 2007).

Initiation of sinusoidal growth was influenced by the rigidity of the agar substrate and the presence of an additional carbon source but sinusoidal wavelength and amplitude were not affected by these parameters. Changes in sinusoidal hypha morphology were only observed in *C. albicans* mutant strains. Chs3 is responsible for synthesis of short, rod-like chitin fibrils in the lateral wall of *C. albicans* (Lenardon *et al*., 2007). The induction rate of sinusoidal hypha formation in *chs3*Δ was the same as the control strain but the amplitude of its sinusoidal hyphal compartments was greater. This effect was not observed when hyphae were treated with Calcofluor White, which destabilizes hydrogen bonding between chitin fibrils (Elorza *et al*., 1983). Changes in sinusoidal hypha morphology were also observed in the *mid1*Δ mutant. In the absence of *PMR1*, hyphae grew in straight trajectories under conditions that would otherwise induce sinusoidal growth. Re-integration of a single copy of *PMR1* partially overcame the block to sinusoidal hypha initiation. Hyphae of the re-integrant strain formed sinusoidal hyphae with a shorter wavelength than the control strain. The switch to sinusoidal growth therefore appears to involve calcium signalling and to be affected by processes that may affect the normal distribution and concentration of intracellular calcium.

The mechanisms underlying the formation of regular sinusoidal curves and helices are unknown but, in addition to substrate rigidity, low nutrient availability is a key inducer in fungi that naturally exhibit this behaviour, such as *C. albicans*, *S. fimicola* and *Saprolegnia ferax* (Madelin *et al*., 1978; Kaminskyj and Heath, 1992; Sherwood-Higham *et al*., 1995). In other filamentous fungi, helicity has been observed as a result of mutations that lead to defective delivery of membranous organelles to the hyphal tip, or alter the size, position or mobility of the Spitzenkörper (Wu *et al*., 1998; Riquelme *et al*., 2000; Takeshita *et al*., 2008). The positioning of the Spitzenkörper within the hyphal tip is thought to predict the site of new tip growth (Riquelme *et al*., 1998). The remarkable regularity of the sinusoidal curves formed by *C. albicans* hyphae, together with the observed tendency for septa to be localized at alternate apices (Fig. 5), suggest that the Spitzenkörper oscillates in two dimensions across the longitudinal growth axis in a cell cycle-dependent manner. In the green alga, *Chara globularis*, relocalization of Ca^2+^ channels and the tip-high calcium gradient are suggested to precede and mediate changes in tropic growth direction by displacing the Spitzenkörper (Braun and Richter, 1999). Our observation that disruption of calcium homeostasis by manipulation of Ca^2+^ availability or by Ca^2+^ channel deletion severely affects the normal initiation and morphology of sinusoidally curved growth in *C. albicans* hyphae is consistent with this view.

The function and relevance of sinusoidal growth in fungal hyphae is not known. It has been proposed that such directional growth offers greater exploration of the environment than unidirectional growth (Sherwood-Higham *et al*., 1995; Garrill, 2000). In the pathogenic oomycete, *S. ferax,* helicity and other changes in hyphal morphology are affected by the presence of bacterial polysaccharides but the relevance of this *in vivo* is unclear (Kaminskyj and Heath, 1992). *C. albicans* is an opportunistic human pathogen and yeast cells colonize endothelial and epithelial surfaces prior to the ensuing process of tissue invasion. To our knowledge, hyphae have not been observed to produce regular sinusoidal or helical growth in tissue sections derived from biopsies or from mouse models of infection, but adhered hyphae have been imaged undergoing directional changes in order to penetrate the underlying semisolid tissue in a reconstituted model of oral epithelium (Zakikhany *et al*., 2007). As unidirectional hyphal growth would not represent the optimal strategy for tissue penetration and ramification, it is possible that partial helix formation in the form of hypha tip rotation is a mechanism that enables hyphae to facilitate infiltration into a relatively immunologically protected and nutrient-rich host tissue.

## Experimental procedures

### Strains, media and growth conditions

The *C. albicans* strains used in this study are listed in Table 1. Strains were maintained and grown overnight at 30°C in YPD [1% w/v yeast extract (Oxoid, Unipath, Basingstoke, UK), 2% w/v mycological peptone (Oxoid), 2% w/v glucose (Sigma, Poole, UK)], solidified as appropriate with 2% agar (Oxoid). Hyphae were grown on solid medium containing 20% (v/v) FBS (Biosera, Ringmer, Sussex, UK) in ddH_2_O, solidified using 1%, 2%, 4% or 6% (w/v) purified agar (Oxoid) (see *Results*). Solid medium was supplemented with 2% (w/v) glucose, maltose or sorbitol (Fisher Scientific, Loughborough, UK), 1.5 mM BAPTA (a Ca^2+^ chelator) tetrapotassium salt (Sigma) or 1.5 mM CaCl_2_ (Sigma), as required. Alternatively, the medium was supplemented with 50 μg ml^−1^ Calcofluor White, 200 μg ml^−1^ Congo Red, 500 μM gadolinium or 250 μM verapamil (Sigma). The Ca^2+^ concentration of 20% fetal calf serum (FCS) was determined as 0.7 mM using a QuantiChrom Calcium Assay Kit (DICA-500) (BioAssays, Hayward, USA) according to the manufacturer's instructions.

**Table 1:** *C. albicans* strains used in this study.

Strain	Genotype	Description	Reference
NGY152	CAI4/CIp10-*URA3*	Control strain	Brand *et al*. (2004)
3153A	Wild type	Clinical isolate	Mackenzie and Odds (1991)
NGY166	*cch1*Δ	Mutant lacking a plasma-membrane, voltage-gated calcium channel	Brand *et al*. (2007)
NGY167	*mid1*Δ	Mutant lacking a plasma-membrane, stretch-activated calcium channel	Brand *et al*. (2007)
NGY368	*cch1*Δ/*mid1*Δ	Mutant lacking the Cch1-Mid1 plasma-membrane calcium channel complex	Brand *et al*. (2007)
NGY468	*mid1*Δ+*MID1*	*mid1*Δ mutant with 1 copy of *MID1* re-integrated at the *RPS1* locus	Brand *et al*. (2007)
NGY355	*pmr1*Δ	Golgi-ATPase mutant, partially deficient in *O*- and *N*-glycosylation	Bates *et al*. (2005)
NGY356	*pmr1*Δ+*PMR1*	*pmr1*Δ mutant with 1 copy of *PMR1* re-integrated at the *RPS1* locus	Bates *et al*. (2005)
MKY380	*crz1*Δ	Mutant lacking the calcium-dependent, calcineurin-activated transcription factor	Karababa *et al*. (2006)
MKY381	*crz1*Δ+*CRZ1*	*crz1*Δ mutant with 1 copy of *CRZ1* re-integrated at the *RPS1* locus	Karababa *et al*. (2006)
CACB8B-5	*chs3*Δ	Mutant lacking the chitin synthase responsible for the majority of chitin in the lateral cell wall	Bulawa *et al*. (1995)
NR3	*fks1*Δ	Golgi ATPase mutant, partially deficient in *O*- and *N*-glycosylation	Douglas *et al*. (1997)
HMY5	*mns1*Δ	Mutant lacking outer-chain *N*-glycans and phosphomannan	Mora-Montes *et al*. (2007)
HMY6	*mns1*Δ+*MNS1*	*mns1*Δ with 1 copy of *MNS1* re-integrated at the *RPS1* locus	Mora-Montes *et al*. (2007)
NGY337	*mnt1/2*Δ	*O*-glycosylation double mutant lacking terminal mannan residues	Munro *et al*. (2005)
NGY335	*mnt1/2*Δ+*MNT1*	*mnt1/2*Δ double mutant with 1 copy of *MNT1* re-integrated at the *RPS1* locus	Munro *et al*. (2005)

### Characterization of sinusoidal growth in hyphae

Overnight cultures of yeast cells were harvested by centrifugation and washed twice in ddH_2_O. Cells were diluted 1/2500 and 200 μl was spread onto the surface of 20% (v/v) FCS solid medium and incubated at 37°C for 10 h or until the mean length of hyphae was > 80 μm. Coverslips were placed on the surface of the medium and hyphae were viewed by light microscopy using an Olympus BX50F4 microscope fitted with an Olympus DP11-P camera. The number of primary hyphae over > 80 μm in length that formed two or more consecutive sinusoidal waves was expressed as a percentage of all primary hyphae formed. The morphology of sinusoidal hyphae was characterized by analysing the wavelength (λ) (distance between helical apices), amplitude (A) and cell length (length of cell compartment bounded by two septa) using Openlab 5.0 software (Improvision, Coventry, UK). More than 100 hyphae were observed per strain per experiment. Each experiment was performed on three or more independent occasions. Statistical analysis was performed using the Dunnett's *t*-test as part of SPSS statistical software (Woking, UK).

### Fluorescence microscopy

Yeast cells were adhered to poly-l-lysine slides in sterile water for 20 min and non-adhered cells washed off. Hyphae were induced by incubation in 1% (v/v) serum at 37°C for 5 h. The supernatant was removed and hyphae pulsed with 1 μl of FM4-64 (from 16 mM in DMSO stock) in 1 ml of the retained supernatant by incubation at 37°C for 45 min in the dark. Slides were rinsed with water and incubated for a further 2 h in the dark at 37°C in fresh medium. After further rinsing, 10 μl Calcofluor White (from 10 mg ml^−1^ stock) in 990 μl ddH_2_O was added and the slide viewed under an Axioplan 2 microscope (Carl Zeiss Ltd, UK) fitted with an ORCA-ER camera (Hamamatsu Photonics, Hamamatsu, Japan). Images were captured using Openlab 5.0 software (Improvision, Coventry, UK).

### Confocal microscopy

Cells were grown on solid medium (as above) in 60 mm Petri dishes. Hyphae were stained by the addition of the amphiphilic styryl dye FM4-64 (16 μM in 3 ml dH_2_O from 16 mM stock in DMSO) (Molecular Probes Europe BV) and incubation in the dark for 30 min. Dishes were positioned on the stage of a Zeiss LSM510 META confocal microscope and 3 ml dH_2_O added to the staining solution above the agar. Hyphae were viewed with a ×63 (NA 0.9) water immersion objective using an Argon 488 laser. Images were captured and manipulated using Zeiss LSM510 software (Ver 3.2).
